# Weighted Decoding for the Competence Reliability Problem of ECOC Multiclass Classification

**DOI:** 10.1155/2021/5583031

**Published:** 2021-10-25

**Authors:** Lei Lei, Yafei Song

**Affiliations:** ^1^College of Information and Navigation, Air Force Engineering University, Xi'an 710077, China; ^2^College of Air and Missile Defense, Air Force Engineering University, Xi'an 710051, China

## Abstract

Error-Correcting Output Codes has become a well-known, established technique for multiclass classification due to its simplicity and efficiency. Each binary split contains different original classes. A noncompetent classifier emerges when it classifies an instance whose real class does not belong to the metasubclasses which is used to learn the classifier. How to reduce the error caused by the noncompetent classifiers under diversity big enough is urgent for ECOC classification. The weighted decoding strategy can be used to reduce the error caused by the noncompetence contradiction through relearning the weight coefficient matrix. To this end, a new weighted decoding strategy taking the classifier competence reliability into consideration is presented in this paper, which is suitable for any coding matrix. Support Vector Data Description is applied to compute the distance from an instance to the metasubclasses. The distance reflects the competence reliability and is fused as the weight in the base classifier combination. In so doing, the effect of the competent classifiers on classification is reinforced, while the bias induced by the noncompetent ones is decreased. Reflecting the competence reliability, the weights of classifiers for each instance change dynamically, which accords with the classification practice. The statistical simulations based on benchmark datasets indicate that our proposed algorithm outperforms other methods and provides new thought for solving the noncompetence problem.

## 1. Introduction

Multiclass classification has been an open issue in machine learning and many solutions are possible. As a “divide-and-conquer” framework, Error-Correcting Output Codes (ECOC) [1, 2] can realize the class decomposition and dichotomize ensemble effectively through a binary or ternary matrix, which not only simplifies the complexity of pattern recognition but also enables the classic binary classifiers suitable for multiclass classification. So far, ECOC has been widely applied to biological data recognition [3, 4], disease diagnosis [5–7], military target recognition [8], and intelligent transportation systems [9] with fairly good recognition performance.

The procedures of using ECOC to solve the multiclass problems are usually divided into three steps: coding, base classifier training, and decoding.

The goal of coding is to construct a binary or ternary matrix **M**=(*m*_*ij*_)_*c*×*l*_, *m*_*ij*_ ∈ {1,0, −1}, where the rows hold the codewords and the columns represent the binary splits. The coding methods can be categorized into three kinds based on the existing researches: predefined code, data-dependent code, and dichotomies-based code. Independent of the specific application and instances, the predefined code ignores the potential information of the original classes and confines the improvement of classification performance. The dichotomies-based code involves finding an optimal matrix given a set of binary classifiers, which is proven to be an NP-complete problem by Crammer and Singer [10]. However, the data-dependent code can make the best of the class separability to enhance performance as a whole, which has drawn special attention. The classic data-dependent codes contain Discriminant ECOC [11], Subclass ECOC [12], and HECOC [13]. In order to construct a data-driven matrix and enhance the diversity between dichotomizers, Zhou et al. [14] proposed a Confusion Matrix Superclass ECOC (CMECOC) based on the Fisher criterion. CMECOC used “one-versus-all” method to encode classes between the superclasses to avoid the unnecessary redundancy and “one-versus-one” method among the superclasses to reduce the complexity of decision boundaries. More research studies on data-driven ECOC are available [15–21], all of which promote the ECOC development.

The second step is base classifier training. The most common practice is to train the classifiers (decision functions) with the binary splits. The outputs of the base classifiers are merged in the final decoding step. It is generally acknowledged that there are three categories of decoding strategies according to the existing literatures. The first type is those based on the distance between the predicted codeword and the target codeword. The state-of-the-art decoding strategies of this type contain the Hamming decoding, Euclidean decoding, loss-based decoding, and so on. Zhou et al. [22] proposed a new weighted decoding by using genetic algorithm to learn the weight coefficient matrix and taking the final generalization error of the ensemble classifiers as fitness function to reduce the error caused by the consistent-diverse balance problem. However, the predicted codewords of the base classifiers are hard (either 1 or −1), which may be not suitable for the cost-sensitive classification. On this account, Lei et al. [23] introduced the reject option into coding phase to extend a binary-symbol output to a three-symbol one and used the modified Hamming decoding strategy for decoding.

The second type is those based on class posterior probability estimation, which estimates the class membership based on the decomposition framework of ECOCs. Suppose that the base classifier *h*(*x*) outputs the class probability for a given test sample **x**; then the probability vector can be obtained as follows:

(1) $$\begin{array}{c} Y=\left(P\left(h_{1}\left(x\right)=1\right),P\left(h_{2}\left(x\right)=1\right),\ldots ,P\left(h_{L}\left(x\right)=1\right)\right), \end{array}$$

where *L* is the number of **M** columns. Then the relationship between the coding matrix, probability vector, and the posterior probability **p**=(*P*(*c*_1_*| ***x**),…, *P*(*c*_*k*_*| ***x**)) can be described as

(2) $$\begin{array}{c} M^{T}p=Y, \end{array}$$

where *k* is the number of classes. Many research works have been done based on the equation. Zhou et al. [24] put forward a variation of the product rule and a linear rule based on posterior probabilities for ternary ECOC. The authors refined the decomposition process of the probability to get smoother estimates in the product rule and extend the linear rule in binary ECOC to ternary ones. Simeon et al. [25] designed the reject rules in decoding with an external and internal approach. The third type is those based on the analysis of the pattern space [26].

As Prior and Windeat et al. [27] pointed out, it was exactly the diversity inherent to ECOC decomposing framework that makes multiclass classification based on ECOC efficient. It is known that, due to the third symbol, ternary ECOC becomes more versatile and robust. However, when using ternary ECOC, we usually need to face the contradiction between a base classifier and the testing samples whose real class is not used to learn the classifier. We call this contradiction the noncompetence problem. On this account, Galar et al. [28] proposed a dynamic classifier selection strategy for the specific one-versus-one matrix that tried to avoid the noncompetent classifiers. The strategy considered the neighborhood of each instance to decide whether a classifier is competent or not. Furthermore, they developed a distance-based combination strategy to reduce the effect the noncompetence contradiction. In the strategy, the degree of the base classifiers' competence reliability was measured with the distance from the instance to each class [29].

However, Galar et al.'s work mainly concentrated the noncompetence problem on the predefined code (one-versus-one scheme) [30]. With the development of coding, most of the ternary ECOC are data-driven matrices with high base classifier diversity and low column redundancy. How to reduce the error caused by the noncompetence contradiction for the data-driven matrices under diversity big enough is the breakthrough of the ECOC classification. To this end, we propose a new weighted decoding strategy by considering the competence reliability (WCR) to decrease the error caused by the noncompetent classifiers. In the proposed algorithm, the SVDD is used to measure the closeness of an instance to each class, which can be regarded as the estimates of the competence reliability of the base classifiers for an instance. Then, we can get the weight coefficient matrix, in which the weights of the competent classifiers are higher and those of the noncompetent ones are lower. In so doing, the influence of the noncompetence contradiction on classification can be weakened or eliminated.

The outline of the paper is organized as follows: a brief introduction of the noncompetence problem and SVDD are discussed in Section 2.  Section 3 focuses on the proposed WCR weighted algorithm. Experiments and results are summarized in Section 4 and Section 5 presents conclusions.

## 2. Noncompetence Problem

According to the pattern recognition theory, a good classification algorithm always demands for the same distribution of training samples and testing samples. Some original classes are denoted by zero when a ternary ECOC is used for classification. The testing samples and training samples for a base classifier may not belong to the same metasubclass. Therefore, a classifier is noncompetent for an instance whose real class does not belong to the metasubclass used to learn the classifier. It is a prior question to estimate whether a base classifier is competent or not for an instance, which is equivalent to the classification problem itself. However, the outputs of noncompetent classifiers are merged in the decoding step equivalently. The existing data-driven matrices possess big diversity and fewer columns. The bias induced by the noncompetent classifiers may yield a decisive error for the final decoding results.

**Figure 1:**
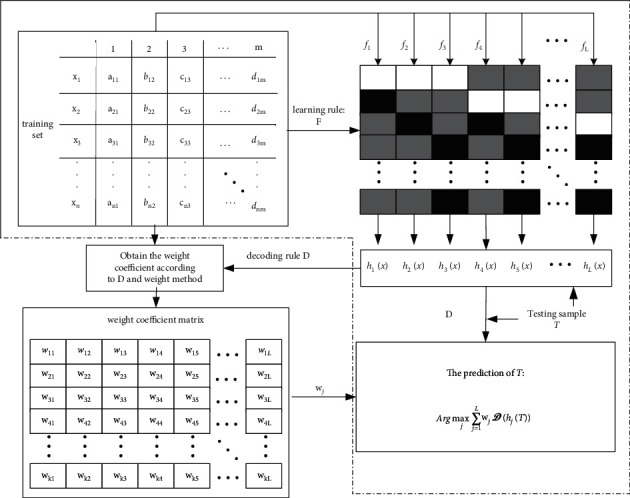
The ECOC framework based on WCR weighted decoding.

Taking the analysis above into consideration, to reduce the error induced by the noncompetent classifiers, it is crucial to determine how to estimate the competence reliability of a classifier for a testing instance. In order to achieve this goal, we present a new WCR decoding strategy to balance the noncompetence contradiction. As for the weighted decoding, Hüllermeie and Vanderlooy [31] provided a sound theoretical demonstration from the perspective of the optimal adaptive voting strategy. Zhou et al. [22] also acknowledged the significance of the weighted method and advocated that different weighted schemes led to different decoding results and were more important than decoding strategies themselves under some circumstances. The proposed WCR decoding can estimate the competence reliability by learning the weight coefficient matrix based on the column outputs. The ECOC framework based on WCR weighted decoding is described in Figure 1.

**X** is the training set with *m* dimensions and *n* samples. *f* denotes the base classifier. *h*(*·*) is the base classifier output. (*w*_*ij*_)_*k*×*L*_ is the weight coefficient matrix, in which *k* and *L* are the numbers of the classes and columns, respectively. The traditional ECOC classification is shown in the dotted box. *h*(*·*) can be trained with the binary splits based on the ternary matrix where the white rectangles represent 1, the black ones represent −1, and the gray ones are 0, which means the corresponding classes are not used to train the base classifier. The weight coefficient matrix can be obtained according to the decoding rule **D** and weight method. The final results can be achieved by the following formula:

(3) $$\begin{array}{c} \text{Arg}\underset{j}{\max}\sum_{j=1}^{L}w_{j}D\left(h_{j}\left(T\right)\right). \end{array}$$

The key of WCR ECOC is how to calculate the weight coefficient matrix which reflects the competence reliability of the classifier for an instance. As we know, the closer an instance to a class, the more likely it belongs to, the more competent the base classifier trained with the corresponding class. So the question is how to measure the distance between an instance and each class.

First proposed by Tax and Robert [32], SVDD is an effective and commonly used single-class classification mechanism. SVDD obtains a spherically shaped boundary around a dataset **X**={**x**_1_, **x**_2_,…, **x**_*N*_} ⊂ **R**^*m*^ and can be made flexible by using kernel functions to project the original nonlinear data to high-dimensional feature space. Featured by the center **o** and radius*r* > 0, the hypersphere covers all of the target data and superfluous space as little as possible, which can be used to detect nontarget data or outliers to realize two-class classification.

The hypersphere can be obtained by minimizing *r*^2^ with the constraint ‖**x**_*i*_ − **o**‖^2^ ≤ *r*^2^. In order to make the optimal zone more compact, according to the kernel projection notation, the low-dimensional input space *F* can be reflected into high-dimensional feature space *H* by nonlinear function Φ. The inner product operation in *H* can be replaced by the kernel function satisfied with Mercer constraints, which means finding the kernel function *K*(*x*, *y*) subjected to *K*(*x*, *y*)=〈Φ(*x*), Φ(*y*)〉; after that, fix the hypersphere in *H* [33]. Allowing for the possibility of outliers, the distance from **x**_*i*_ to *ο* should not be strictly smaller than *r*^2^, but a larger distance should be penalized. Therefore, the slack variable *ξ*_*i*_ ≥ 0 is introduced and the minimization problem can be rewritten as follows:

(4) $$\begin{array}{c} \underset{r}{\min}r^{2}+C\sum_{i}\xi_{i}\\ \text{s.t}\;{\left\|x_{i}-o\right\|}^{2}\leq r^{2}+\xi_{i}\text{,}\;\xi_{i}\geq 0,\;i=1,2,\ldots ,N. \end{array}$$

Parameter *C* controls the trade-off between *r* and the number of outliers. The optimal problem can be solved by changing into a dual problem with Lagrange multipliers:

(5) $$\begin{array}{c} \max\sum_{i}\alpha_{i}K\left(x_{i},x_{i}\right)-\sum_{i,j}\alpha_{i}\alpha_{j}K\left(x_{i},x_{j}\right)\\ \text{s.t.}\;0\leq \alpha_{i}\leq C,\sum_{i}\alpha_{i}=1,j=1,2,\ldots ,N. \end{array}$$

Objects **x**_*i*_ with 0 < *α*_*i*_ ≤ *C* are called the support vectors of the SVDD.

For a testing sample **x**,

(6) $$\begin{array}{c} f\left(x\right)={\left\|x-o\right\|}^{2}=K\left(x,x\right)-2\sum_{i=1}^{N}\alpha_{i}K\left(x,x_{i}\right)+\sum_{i}^{N}\sum_{j}^{N}\alpha_{i}\alpha_{j}K\left(x_{i},x_{j}\right). \end{array}$$

If ‖**x** − **o**‖^2^ ≤ *r*^2^, object **x** is recognized as the target class, and vice versa.

## 3. Weighted Decoding for the Competence Reliability Problem

### 3.1. Construction of Weight Coefficient Matrix

Consider a *k*-class classification case and the training dataset {*X*_1_,…, *X*_*k*_}, in which *X*_*i*_={**x**_1_, **x**_2_,…, **x**_*N*_*i*__}, *i*=1,…, *k*, and *N*_*i*_ is the number of each class. *N*=∑_*i*=1_^*k*^*N*_*i*_.

The first step of the WCR weighted decoding is to use SVDD to compute the distance from an instance to each class. The SVDD hyperspheres *S*={*S*_1_,…, *S*_*k*_}={(*r*_1_, **o**_1_), (*r*_2_, **o**_2_),…, (*r*_*k*_, **o**_*k*_)} for each class are achieved with *X*_*i*_, respectively, by using the kernel function. The Euclidean distance between an instance and the *i*^*th*^ class is computed with $d_{i}=\sqrt{\left\|x-o_{i}\right\|}$. The distance vector can be obtained as **D**=(*d*_1_, *d*_2_,…, *d*_*k*_). We may make the following assumption: an instance belongs to a class if the distance is equal to or smaller than the radius, and vice versa. $\bar{D}=\left(d_{1}/r_{1},d_{2}/r_{2},\ldots ,d_{k}/r_{k}\right)$ for belonging preference relation is identified by normalizing the distance vector. From the analysis above, we can see that if *d*_*i*_/*r*_*i*_ is not more than 1, the instance is likely to belong to class_*i*_. The smaller *d*_*i*_/*r*_*i*_, the larger the probability, while the larger the ratio, the smaller the probability.

Given a ternary matrix **M**=(*m*_*ij*_)_*c*×*l*_,  *m*_*ij*_ ∈ {1,0, −1}, if *M*_*ij*_=0, *class*_*i*_ is omitted in the *j*^*th*^ base classifier training. If an instance belongs to class_*i*_, then the base classifier is noncompetent for the instance, whose outputs have little guidance for classification prediction. Therefore, the influence of the *j*^*th*^ classifier should be weakened when the instance is classified. The WCR decoding relearns the weight of the base classifier based on competence reliability by using the following formula:

(7) $$\begin{array}{c} w_{ij}=\frac{d_{i}}{r_{i}},\text{ if }M\left(i,j\right)=0. \end{array}$$

It is worth noting that more than one class will be ignored during the training, so the final weight of base classifier is obtained:

(8) $$\begin{array}{c} w_{j}=\sum_{i=1,M\left(j,i\right)=0}^{N_{i}}\frac{d_{i}}{r_{i}}. \end{array}$$

From equation (7), we may draw the following conclusions.

Given an instance, whose real class label is class_*i*_, the distance from the instance to class_*i*_ is the shortest. If class_*i*_ is ignored during the base classifier training, the weight of the corresponding base classifier in the decoding phase becomes smaller because *d*_*i*_/*r*_*i*_ ≤ 1 and it is noncompetent. The more the classes ignored, the smaller the weight and the less competent the base classifier. According to the aforementioned considerations, the WCR weighted strategy is listed in Algorithm 1.

In order to elaborate the proposed method, the Balance benchmark dataset, 625 by 4 dataset with 3 classes from the University of California at Irvine (UCI) repository, is taken as an example. The numbers of the training and testing sets in one of the cross-validation folds are 499 [39 230 230] and 126 [58 10 58], respectively. The hypersphere configurations obtained by the proposed method during the training phase are indicated in Table 1.

In Table 1, *σ* is the value of radial basis function used in SVDD, *α*_*i*_ is the solution to equation (4), and threshold is the radius range from the support vectors to the hypersphere center.

Consider a testing instance [1 5 2 4] belonging to class_1_. According to Algorithm 1, the distance vector **D** is (0.5417, 0.6978, 0.6519). If the ternary matrix is one-versus-one matrix, the weights of three base classifiers are 0.6519/0.4652=1.4013, 0.6978/0.4482=1.5569, and 0.5417/0.6003=0.9024, respectively. The weight of the third base classifier is the smallest because class_1_ does not take part in the base classifier training, so the third base classifier is noncompetent for the testing instance and the weight is smaller than the other two competent classifiers.

After constructing the weight coefficient matrix, we can get the final results based on decoding strategy.

(9) $$\begin{array}{c} \text{Arg}\underset{j}{\max}\sum_{j=1}^{L}w_{j}D\left(h_{j}\left(T\right)\right). \end{array}$$

### 3.2. Computational Complexity of the Proposed Weighted Decoding Strategy

Compared to the traditional ECOC classification, the time of our proposal is mainly consumed in constructing SVDD hyperspheres, which needs to solve the quadratic programming problem. The testing set has a complexity of Ο(*m∗N*) to compute the distance from an instance to the center, where *N* is the number of samples and *m* is the dimension of attributes. Therefore, the complexity has not obviously increased. The computation time and storage are acceptable.

The approach of the weighted decoding for competence reliability comes into being. Next, we will validate the performance of its application in the classification through experiments.

## 4. Experiments and Comparisons

### 4.1. Experiment Data

In this section, we validate the proposed algorithm by using benchmark datasets. The characteristics of the UCI datasets are listed in Table 2. Certain UCI datasets are normalized by deleting the small samples. In the meanwhile, the principal component analysis is applied to reduce the dimensionality to promote classification speed.

### 4.2. Experimental Design

Three kinds of experiments are executed for validating the proposed method in this paper.

Firstly, the benchmark datasets are used to evaluate the classification performance of the WCR decoding by comparing the results with four classic decoding strategies: Hamming decoding (HD), inverse Hamming decoding (IHD), linear loss function-based decoding (LLB), and exponential loss function-based decoding (ELB). These strategies all belong to the first type of the decoding category.

Secondly, we compare the results of the weighted Hamming and LLB decoding based on error rate, class separability, and genetic algorithm [26], respectively. Five different ternary matrices, one-versus-one, spares random, subclass ECOC [12], HECOC based on SVDD [30], and CMECOC [22], are adopted in the experiments. When selecting the random code, we pick up the wanted matrix at random in the matrix set of 2000 sparse random matrices (whose probability of each code word is *p*(−1)=1/3, *p*(0)=1/3, and *p*(+1)=1/3).

Finally, the WCR weighted method is compared to the distance-based relative competence weighting (DRCW) algorithm based on *k*-nearest neighbors [29] to evaluate the efficiency of estimating the competence reliability and solving the noncompetence problem. The one-versus-one coding matrix is adopted for consistency.

Decision tree and support vector machine (SVM) base classifiers are adopted in comparison, whose parameter configuration is shown in Table 3.

To evaluate the performance of different experiment results, we apply stratified tenfold cross-validation and test for the confidence interval at 95 percent with a two-tailed test if the number of the pieces of sample data is larger than 500. Otherwise, we apply stratified fivefold cross-validation [34] and the calculation formula is given by

(10) $$\begin{array}{c} \frac{\left|\bar{x}-\mu \right|}{\sigma /\sqrt{n}}\geq t_{0.025}\left(n-1\right), \end{array}$$

where *μ* and *σ* indicate the mean and variance, respectively, *n* is the number of fold validations, and *t*_0.025_(4)=2.7764 and *t*_0.025_(9)=2.2622.

### 4.3. Experimental Results and Analysis

#### 4.3.1. Comparison of Classification Performance

**Figure 2:**
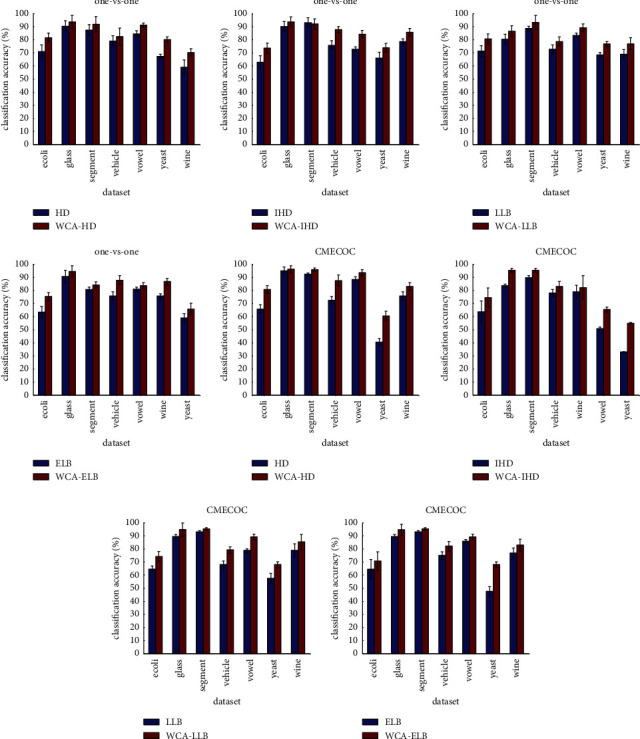
Comparison of four original decoding strategies and the corresponding WCR mechanism.

We compare the classification results of the WCR decoding with those of the original decoding strategies based on one-versus-one matrix and CMECOC. The former belongs to the predefined-based matrix and the latter belongs to the data-driven type.  Figure 2 shows the classification accuracy of these decoding strategies mentioned before based on decision tree classifier.

From the results, we can see that the WCR decoding mechanism outperforms the original decoding ones on most of the datasets, which proves that the weighted decoding for competence reliability problem indeed has a positive influence on the classification performance regardless of the coding matrix. From the other way around, the idea of considering the noncompetence contradiction for the base classifier has practical significance. Effective and feasible, the WCR decoding possesses a remarkable promotion by evaluating the distance from an instance to each class based on SVDD and shifting the distance information into weight to merge in decoding phase.

**Figure 3:**
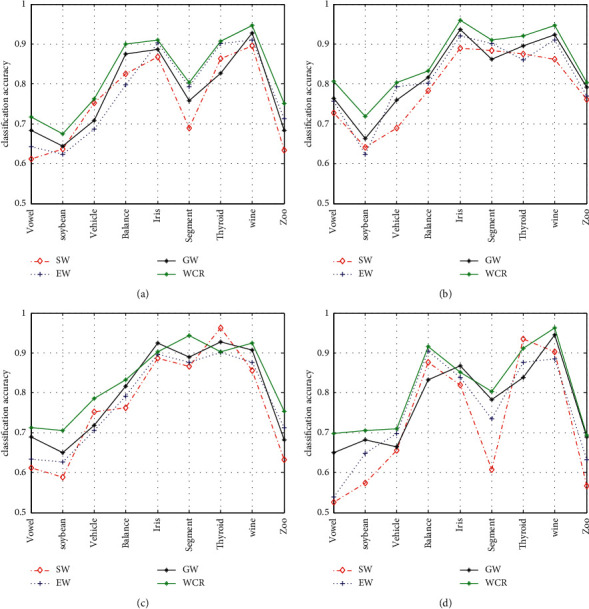
Classification accuracy of four different weighted decoding strategies. (a) Different weighted methods based on Hamming decoding and OVO. (b) Different weighted methods based on LLW decoding and OVO. (c) Different weighted methods based on Hamming decoding and sparse matrix. (d) Different weighted methods based on LLW decoding and sparse matrix.

Figure 3 shows the classification accuracy of four different kinds of the weighted decoding strategies based on class separability (SW), error rate (EW), genetic algorithm (GW), and competence reliability (WCR), respectively.

**Figure 4:**
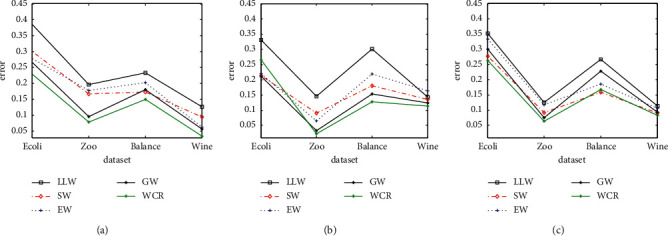
Classification error of certain datasets based on three data-driven matrices and LLW decoding strategy. (a) Different weighted decoding based on SECOC and LLW decoding. (b) Different weighted decoding based on CMECOC and LLW decoding. (c) Different weighted decoding based on HECOC and LLW decoding.

In order to evaluate the performance comprehensively, Figure 4 shows the classification error for three data-driven matrices based on linear loss-based decoding and the corresponding weighted mechanism. From Figures 3 and 4, we can see that the green line representing the WCR strategy is on the top for accuracy and on the bottom for error on most of the datasets, illustrating that the decoding strategy based on the competence reliability performs better than the rest.

Different from the weighted decoding strategies based on error rate, class separability, and genetic algorithm, in which the weights for base classifier are static, the weights in WCR mechanism dynamically change along with the distance from the testing instances to each class. In the meanwhile, the weights in classic methods are obtained in the testing step. The weights in WCR are got throughout the testing phase, which is much close to the real classification practice.

It is worth noting that some other weighted decoding algorithms perform the best sometimes. This can be explained by the fact that the corresponding dataset has a balanced distribution. Compared to the WCR decoding algorithm, there is only a little difference in the weight matrix got by error rate and class separability, which can still achieve the sound classification performance under certain situation suitable for their starting points.

**Figure 5:**
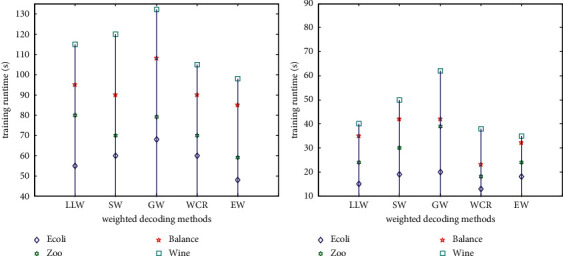
The training and testing runtime of different weighted decoding methods.

Figure 5 shows the runtime of different weighted decoding strategies. The computation complexity of WCR focuses on the hypersphere construction. Once the hyperspheres are built, short testing time is required, so the computation complexity is acceptable. In the meanwhile, the weighted decoding based on GW possesses the longest runtime because the weights are obtained by solving an optimal problem. The runtime of weighted decoding based on error rate is the shortest, which can be explained by the fact that the weights based on error rate can be got directly from the training accuracy.

#### 4.3.2. Comparison of Competence Reliability Evaluation

In order to validate the efficiency of our proposal further, the WCR mechanism based on SVDD is compared with the DRCW-OVO based on *k*-nearest neighbors. The coding is one-versus-one matrix, and SVM and decision tree are chosen as base classifiers, where *k*=3*∗*number of class.  Table 4 lists the accuracy of two methods of evaluating competence reliability, where the bold face denotes the best classification accuracy.

From Table 4, we can see that the accuracy of the weighted decoding based on WCR wins over DRCW twelve times in total 32-time experiment.

In order to get the statistical comparison, we calculate the average ranks as 1.75 and 1.25 for DRCW and WCR, respectively. The Nemenyi test [35] is used further to test the significant difference between the two methods. The critical difference value $\text{CD}=q_{a}\sqrt{k\left(k+1\right)/6J}=q_{0.05}\sqrt{2\times 3/6\times 16}=0.49$, where *q*_*a*_ is the Studentized Range Statistic and *k* and *J* are the number of methods to be compared and experiment time for each method, respectively. We can see that the average rank of WCR and the difference are both larger than CD, which means the difference between the two methods is significant and the performance of WCR with lower rank is better.

This corroborates the efficiency of WCR in dealing with the noncompetence problem. In the contrast with *k*-nearest neighbors method, the distance obtained by SVDD hyperspheres is more comprehensive for the reason that it takes all the class samples into consideration. In summary, the idea of the weighted decoding based on WCR provides new sparks and thought for solving noncompetence problem.

## 5. Conclusion

How to estimate the competence reliability of base classifiers is a new issue for ternary ECOC classification. In order to reduce the error caused by the noncompetent classifiers, a new WCR weighted decoding is proposed in this paper. To achieve the goal, the SVDD hyperspheres are used to measure the distance from an instance to be classified to each class. Then the distance is applied as the weight to fuse in the decoding step to make the final decision. In so doing, the classifier competence reliability is evaluated quantitatively by the weights. In the meanwhile, the influence of the competent classifiers on classification is reinforced and that of the noncompetent ones is weakened. The experimental results prove the promotion of the WCR weighted decoding for classification. Roughly speaking, the proposed algorithm provides new possibilities for the noncompetence contradiction solutions. How to deal with the outlets for subclass separation and data uncertainty [36] is the next research direction.

## Figures and Tables

**Algorithm 1:**
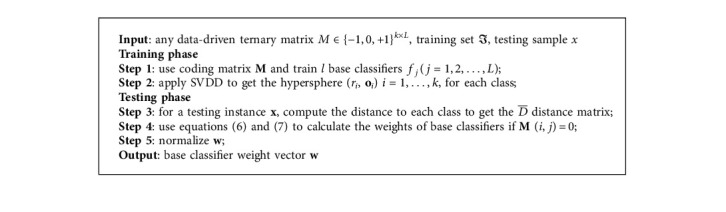
The weight coefficient matrix construction based on SVDD.

**Table 1:** Hypersphere configuration for each class.

	Class_1_	Class_2_	Class_3_
*σ*	5	5	5
*α*_*i*_	8 × 1	30 × 1	30 × 1
Threshold	0.6003	0.4486	0.4653
Support vectors	8 × 4	30 × 4	30 × 4

**Table 2:** The description of benchmark datasets.

Dataset	Cases	Classes	Atts	Features		
				C	B	N
Ecoli	336	8	7	7	—	0
Glass	214	6	10	9	—	−1
Iris	150	3	4	4	—	—
Satimage	6435	6	36	36	—	—
Segment	2310	7	19	19	—	—
Vehicle	846	4	18	18	—	—
Vowel	990	11	13	13	—	—
Wine	178	3	13	13	—	—
Yeast	1484	10	8	8	—	—
Zoo	101	7	16	1	15	—

Features: C, continuous; B, binary; N, nominal.

**Table 3:** The parameter configuration.

Algorithm	Parameters
SVM_poly_	*C* = 1.0 Tolerance parameter = 0.001 Epsilon = 1.0*E*−12 Kernel type = polynomial Fit logistic models = true

SVDD [33]	Fracrej = 0.05 Kernel function = RBF Sigma = 5

Treec	Maxcrit = purity Prune = 0 no pruning

**Table 4:** Classification accuracy of DRCW-OVO and WCR.

Dataset	SVM		Treec	
	DRCW	WCR	DRCW	WCR
Ecoli	66.67 ± 2.93	**69.01** **±** **2.73**	**72.91** **±** **0.86**	69.64 ± 1.15
Glass	**97.26** **±** **3.73**	93.47 ± 3.72	90.54 ± 7.08	**92.98** **±** **2.93**
Satimage	85.84 ± 0.54	**86.14** **±** **3.83**	83.93 ± 1.47	**84.01** **±** **0.79**
Segment	93.25 ± 0.90	**94.87** **±** **1.14**	93.94 ± 1.71	**94.68** **±** **1.35**
Vehicle	79.31 ± 3.38	**79.54** **±** **2.87**	65.97 ± 2.62	**67.50** **±** **1.84**
Vowel	**98.69** **±** **0.84**	85.66 ± 3.27	**82.73** **±** **2.92**	82.02 ± 5.41
Yeast	57.34 ± 2.32	**58.48** **±** **2.60**	51.15 ± 3.88	**51.87** **±** **3.02**
Zoo	81.02 ± 4.12	**83.26** **±** **3.89**	71.23 ± 13.91	**74.58** **±** **3.26**

## Data Availability

All data used in this paper can be obtained by contacting the authors of this study.
